# Tobacco Substitutes: Harm Reduction or Smokescreen?

**DOI:** 10.1371/journal.pmed.0040244

**Published:** 2007-07-31

**Authors:** The PLoS Medicine Editors

## Abstract

The*PLoS Medicine* editors discuss whether publishing papers on smokeless tobacco serves a legitimate public health interest.

The editorial this month was triggered by a discussion among the editors at *PLoS Medicine* about whether or not we should, as a medical journal, be publishing papers on the use of smokeless tobacco (snus), the topic of the debate by Gartner, Chapman, and colleagues [1]). At one end of the spectrum of views expressed, one editor argued that we should not give the topic room in the journal at all, because even discussion of the use of snus simply plays into the hands of the tobacco industry, which has a notorious history of doing anything it can to addict people to tobacco. At the other end of the spectrum, another editor pointed out that since snus is associated with less risk to health than cigarettes, a discussion of its use as a harm reduction measure is an appropriate topic for a medical journal. On this topic, the editors simply could not come to an agreement. (Another such topic, incidentally, is that of qualitative research, on which more in future.)

Don't journals have a duty to give all sides of a debate? That was certainly the argument that the *BMJ* put forward when it published a paper that concluded that harm caused by passive smoking had been overestimated [2]. Unfortunately, that paper turned out not to be free of bias and may in fact have been part of the tobacco industry's ongoing attempt to discredit evidence of the harms caused by passive smoking [3]. In trying to be fair, the *BMJ* may have been the victim itself of cynical maneuvering by an industry not known for its interest in playing by the rules in promotion of its products. By publishing papers such as the one on snus, could we be legitimizing a debate that at best can only shift tobacco dependence from one product to another? Should we only publish papers that point out the wrongdoing of the tobacco industry (e.g., [4]) or advocate the abolition of all tobacco products?

No *PLoS Medicine* editor would argue that profiting from an addiction that severely impairs health is ethical, and none of us would dispute that ceasing to use tobacco is better than switching to snus. The issue is whether, in a world where many people die from their tobacco addiction without overcoming it, we should give room to the opinion that switching to snus, although not better than quitting, may be better in terms of short-term health outcomes than continuing to smoke.

In respect for honest differences of opinion, we are ending this editorial without a bottom line. Do papers on smokeless tobacco serve a legitimate public health interest? We'd like to seek the opinions of our readers: what do you think? 3
